# Agnogene Deletion in a Novel Pathogenic JC Virus Isolate Impairs VP1 Expression and Virion Production

**DOI:** 10.1371/journal.pone.0080840

**Published:** 2013-11-12

**Authors:** Laura C. Ellis, Elizabeth Norton, Xin Dang, Igor J. Koralnik

**Affiliations:** 1 Division of Neurovirology, Department of Neurology, Beth Israel Deaconess Medical Center, Harvard Medical School, Boston, Massachusetts, United States of America; 2 Center for Virology and Vaccine Research, Department of Medicine, Beth Israel Deaconess Medical Center, Harvard Medical School, Boston, Massachusetts, United States of America; 3 Harvard Program in Virology, Harvard Medical School, Boston, Massachusetts, United States of America; University of Utah School of Medicine, United States of America

## Abstract

Infection of glial cells by the human polyomavirus JC (JCV) causes progressive multifocal leukoencephalopathy (PML). JCV Encephalopathy (JCVE) is a newly identified disease characterized by JCV infection of cortical pyramidal neurons. The virus JCV_CPN_ associated with JCVE contains a unique 143 base pair deletion in the agnogene. Contrary to most JCV brain isolates, JCV_CPN_ has an archetype-like regulatory region (RR) usually found in kidney strains. This provided us with the unique opportunity to determine for the first time how each of these regions contributed to the phenotype of JCV_CPN_. We characterized the replication of JCV_CPN_ compared to the prototype virus JCV_Mad-1_ in kidney, glial and neuronal cell lines. We found that JCV_CPN_ is capable of replicating viral DNA in all cell lines tested, but is unable to establish persistent infection seen with JCV_Mad-1_. JCV_CPN_ does not have an increased ability to replicate in the neuronal cell line tested. To determine whether this phenotype results from the archetype-like RR or the agnogene deletion, we generated chimeric viruses between JCV_CPN_ of JCV_Mad-1_. We found that the deletion in the agnogene is the predominant cause of the inability of the virus to maintain a persistent infection, with the introduction of a full length agnogene, either with or without agnoprotein expression, rescues the replication of JCV_CPN_. Studying this naturally occurring pathogenic variant of JCV provides a valuable tool for understanding the functions of the agnogene and RR form in JCV replication.

## Introduction

The human polyomavirus JC (JCV) has a circular double stranded DNA genome which can be divided into 3 regions. The early coding region encodes the regulatory proteins small t antigen and large T antigen (T Ag). The late coding region encodes the VP1, VP2 and VP3 structural proteins, and the agnoprotein [1]. The regulatory region (RR) contains the origin of replication, as well as the early and late promoters [2], [3]. While the coding regions are well conserved, the RR is hypervariable, with different sequences being isolated from individuals [1]. Archetype RR, which has one 98-bp element and contains a 23-bp and a 66-bp insert, is generally found in the kidneys or urine of healthy and immunosuppressed individuals [4]. RRs from the brain or CSF of PML patients are generally of the rearranged type, containing two 98-bp tandem repeats with additional mutations, insertions and deletions [1], [2].

JCV is the etiological agent of Progressive Multifocal Leukoencephalopathy (PML), an often fatal demyelinating disease caused by lytic infection of oligodendrocytes by the virus [5]. Infection with JCV is widespread in the population, but remains asymptomatic in healthy people [6], [7]. Development of PML is associated with immune suppression, such as in patients with AIDS [8], organ transplants [9] or hematological malignancies [10]. We have identified two additional syndromes caused by JCV infection in the brain, JCV Granule Cell Neuronopathy (JCV GCN) [11], [12], [13] and JCV Encephalopathy (JCVE) [14]. The viruses isolated from patients with these syndromes contain previously unreported unique mutations. JCV GCN is associated with deletions in the C-terminus of the VP1 protein [15], [16] and JCVE with a deletion in the agnogene [17]. These naturally occurring pathogenic variants provide a unique tool for studying the basic biology of JCV replication and pathogenesis.

JCVE was described in an HIV-negative patient with a history of lung cancer treated with chemotherapy, who presented with cortical lesions, aphasia and progressive cognitive decline. Post-mortem analysis of the brain showed cortical lesions with productive infection of cortical pyramidal neurons [14]. Isolation and sequencing of the JCV DNA present in the brain of this patient identified a virus with an archetype-like RR and a 143 base pair deletion in the agnogene [17]. This virus was named JCV Cortical Pyramidal Neuron 1 (JCV_CPN1_). The deleted agnogene encodes a 10 amino acid truncated peptide. Further analysis found that multiple forms of JCV_CPN_ were present, and that these strains co-existed with a virus containing a full length agnogene. Immunostaining analysis indicated that the majority of the cortical cells infected with JCV contained the truncated form of the agnoprotein [17].

JCV agnoprotein is a highly basic, 71 amino acid, non-essential protein that is expressed late in infection, but not incorporated into virions [18]. It is primarily expressed in the cytoplasm, particularly in the perinuclear region, with a small amount found in the nucleus [19]. Agnoprotein has been shown to form homodimers and oligimers [20]. Agnoprotein contains 3 phosphorylation sites, which can be phosphorylated by protein kinase C [21] and dephosphorylated by protein phosphatase 2A [22]. The phosphorylation state may impact agnoprotein localization [19]. Agnoprotein has been shown to bind to T Ag, down regulating DNA replication [23] and enhancing T Ag origin binding [24]. Agnoprotein may also influence viral gene expression and splicing of viral transcripts [25]. Loss of agnoprotein expression has been associated with loss of early and late mRNA expression [19]. Agnoprotein has also been shown to suppress activity of the late promoter [23], and to interact with the transcription factor YB-1, inhibiting its ability to activate the early and late promoters [26]. Prevention of agnoprotein expression also has been shown to result in decreased levels of T Ag and VP1 protein expression [27]. Viruses lacking agnoprotein are less efficient at packaging DNA and virion formation, with infected cells releasing empty particles [27], [28]. The agnoprotein may also function as a viroporin, aiding in the release of virions from infected cells [29]. These studies have added to our knowledge of agnoprotein function in recent years, but the exact mechanisms by which this protein influences the viral life cycle remain unclear.

In addition to agnoprotein function, the DNA of the agnogene has been shown to contain 3 host cell factor binding sites. Deletion of the agnogene DNA has a greater effect on replication than prevention of agnoprotein expression by mutation of the start codon [30]. In the agnogene deletion present in JCV_CPN_, 1 of the 3 sites is completely deleted, and a second is shortened by 1 nucleotide.

We hypothesized that the agnogene deletion of JCV_CPN_ allowed the virus to infect cortical pyramidal neurons. We used cell culture models to study the replication of JCV_CPN_ compared to the prototype strain JCV_Mad-1_ [31] in different cell types. We generated chimeric viruses of JCV_CPN_ and JCV_Mad-1_, swapping both the agnogene and RR, to determine the specific effects of the agnogene deletion and the archetype-like RR on viral replication in cell culture. In these experiments we characterized the replication of JCV_CPN_ in different cell types, and determined the relative contributions of the agnogene deletion and archetype-like RR to the JCV_CPN_ replication phenotype. Studying these novel naturally occurring changes in JCV_CPN_ provided unique insights into our understanding of the function of the agnogene and RR form in JCV replication.

## Materials and Methods

### Cell Culture

Cos-7 [32], SVG [33] and IMR-32 [34] cells were purchased from the ATCC. Cos-7 cells were maintained in Dulbecco's Modified Eagle Medium (DMEM) supplemented with 10% fetal bovine serum (FBS), Penicillin (500 units/mL) and Streptomycin (500 µg/mL). SVG cells were maintained in Minimum Essential Medium (MEM) supplemented with Sodium Bicarbonate (1.5 g/L), 10% FBS, Penicillin (500 units/mL) and Streptomycin (500 µg/mL). IMR-32 cells were maintained in MEM supplemented with Sodium Bicarbonate (1.5 g/L), Sodium Pyruvate (1 mM), Non-Essential Amino Acids (NEAA) (Invitrogen), 10% FBS, Penicillin (500 units/mL) and Streptomycin (500 µg/mL).

### Plasmids

Construction of the JCV_CPN_ and JCV_Mad-1_ plasmids was previously described by Dang et al. [17]. To generate the agnogene chimeric viruses Mad-1 C-Agno and CPN M-Agno, the agnogene region was excised using the restriction enzymes ApaI and PciI (New England Biolabs), and the digested DNA was run on an agarose gel. The agnogene DNA and the virus minus the agnogene DNA bands were excised and purified using the QIAquick Gel Extraction Kit (QIAGEN). Agnogene DNA segments were ligated into the viral backbone using T4 ligase (New England Biolabs). The resulting plasmids were transformed into TOP10 (Invitrogen) or XL1-Blue cells (Agilent). Plasmid DNA was maxi prepped (QIAGEN), and plasmids were fully sequenced. The RR chimeras were generated with the same protocol, using the restriction enzymes BamHI and PciI (NEB) to excise the RR. Mad-1 Pt and Mad-1 Del plasmids were previously described [30], and obtained as a generous gift from Dr Safak. The agnogene from these viruses was introduced into our JCV_Mad-1_ or JCV_CPN_ plasmid using the ApaI and PciI restriction sites as previously described.

### Transfection

Full-length JCV genomes were digested out of the plasmid backbone using EcoRI (NEB) and run on a 0.8% agarose gel. The 5 kb virus band was purified using the QIAquick Gel Extraction Kit (QIAGEN). Cells were transfected with 1 µg (for IMR-32 and SVG cells) or 2 µg (for Cos-7 cells) of purified JCV DNA using FuGENE6 transfection reagent (Roche or Promega) in 6 well plates. 3 days post-transfection, cells were passaged 1∶3 to T25 flasks, and subsequently every 3–4 days 1∶4 in T25 flasks. At each passage, supernatant was collected and cells were collected, pelleted and stored at −80°C until further analysis.

### DNA Extraction and Quantitative PCR (QPCR)

DNA was extracted from cell pellets and supernatant samples using the QIAamp DNA Blood Mini Kit (QIAGEN). QPCR was performed as previously described [15]. An RNAase P primer/probe set (Applied Biosystems) was used in a multiplex assay with the JCV primer/probe set on cell lysate samples. Copies RNAse P per reaction was determine and divided by 2 to determine the number of input cells for each reaction. Copies JCV/cell was calculated by dividing the copies JCV per reaction by the number of cells per reaction. All samples were run in triplicate.

### RNA Extraction and qRT-PCR

RNA was extracted using the RNEasy Mini Kit (QIAGEN). RNA samples were digested with rDNAse I (Ambion) to remove any contaminating DNA. Reverse transcription was done using the High Capacity RNA-to-cDNA Kit (Applied Biosystems). QPCR was performed on a 7300 Real-time PCR System using Gene Expression Master Mix (Applied Biosystems). For amplification of the early transcript mRNA, a primer probe set spanning the Large T Ag splice site was used. The primers were JCT208F (5′-CATCAGCCTGATTTTGGTACATG-3′, reverse complement of position 4784–4806) and JCT 279R (5′-CCAGGATTCCCATTCATCTGTT-3′, position 4392–4412). The probe used was JCT-232p (6FAM-5′-AAT AGT TCA GAG GTG CCA AC-3′-MGB, reverse complement of position 4419–4426 and 4771–4482). For detection of late transcript mRNA we used the primers JCVP1-745F (5′-GGTGACAACTTATACTTGTCAGCTGTT-3′, position 2213–2239) and JCVP1-812R (5′-TGCTGGGAACCAGACCTGTT-3′, reverse complement of position 2261–2280) and the probe JCVP1-773p (6FAM-5′-ATG TCT GTG GCA TGT TTA-3′-MGB, position 2241–2248). A TATA-box binding protein (TBP) primer/probe set (Invitrogen) was used as the endogenous control for determination of relative quantity by the comparative *C*_T_ method (also known as the ΔΔCt method) [35]. All samples were run in triplicate wells.

### Western Blotting

Cells were lysed in TNN lysis buffer (50 mM Tris-HCl pH 7.5, 150 mM NaCl, 0.1% NP40) with 0.2 mM Na-Orthovanadate and 1% protease inhibitor cocktail for 30 minutes on ice. Samples were centrifuged at 8000 rpm for 4 min to remove cell debris. Laemmli Buffer (Bio-Rad) was added to whole cell lysate. Samples were boiled for 10 min and run on a 10% SDS-PAGE gel in Tris/Glycine/SDS running buffer (Bio-Rad). Samples were transferred for 2 hours at 150 mAmps to a nitrocellose membrane in Transfer Buffer (Tris/Glycine with 20% methanol) or to a nitrocellulose membrane using the iBlot system (Invitrogen). Membranes were blocked using 5% milk in PBST and incubated overnight at 4°C with VP1 antibody pAB597 (1 mg/mL) diluted 1∶1000 or loading control anti-alpha tubulin [DM1A] (abcam) in 2% milk in PBST. An HRP conjugated goat-anti mouse IgG secondary antibody (Bio-Rad) was used and detection was done with ECL Plus reagent (Thermo Scientific). Signal was detected on film.

### Flow Cytometry Analysis of JCV-Positive Cells

Cells were trypsinized, collected and washed with 2% FBS in PBS. Cells were passed through a 30 µm filter (Miltenyi Biotec) and incubated for 20 minutes at 4°C with Aqua Amine to stain dead cells, and then washed with PBS. Cells were fixed with Cytofix/Cytoperm (BD Biosciences) for 20 minutes at 4°C then washed with PBS. Staining with the primary antibody PAB597 (mouse monoclonal anti-VP1) in Perm/Wash Buffer (BD Biosciences) was done for 2 hours at 4°C. Cells were washed with Perm/Wash Buffer, then PBS and then stained with Alexa 488 conjugated Anti-Mouse IgG (Invitrogen) secondary antibody in Perm/Wash Buffer for 1 hour at 4°C. Cells were washed, and then analyzed using a BD LSR II Flow Cytometer (BD Biosciences).

### JCV Infectivity Test

Cos-7 cells were plated in 6 well plates at low density and allowed to adhere overnight. Cell free supernatant collected from transfected cells was put on the Cos-7 cells, and incubated 2 hours at room temperature with rocking. Supernatant was not digested with DNAse to remove free JCV DNA, as previous experiments have shown that digesting with DNAse does not affect the levels of JCV DNA detected in the supernatant by qPCR, or the percentage of cells subsequently infected from the supernatant (data not shown). The infection was allowed to proceed for 7 days. Cells were then collected and analyzed for either VP1 expression by flow cytometry or JCV DNA by QPCR as previously described.

### Statistical Analyses

Analyses were done using SAS Software version 9.3. Data was tested for normality before statistical analysis. P-values for DNA levels were determined using the non-parametric Wilcoxon Rank Test. P-values for mRNA levels were done using univariate analysis, and taking the student's t p value. Flow cytometry p-values were determined using Student's t test.

## Results

### JCV_CPN_ has a 143 base pair deletion in the agnogene and an archetype-like RR

**Figure 1:**
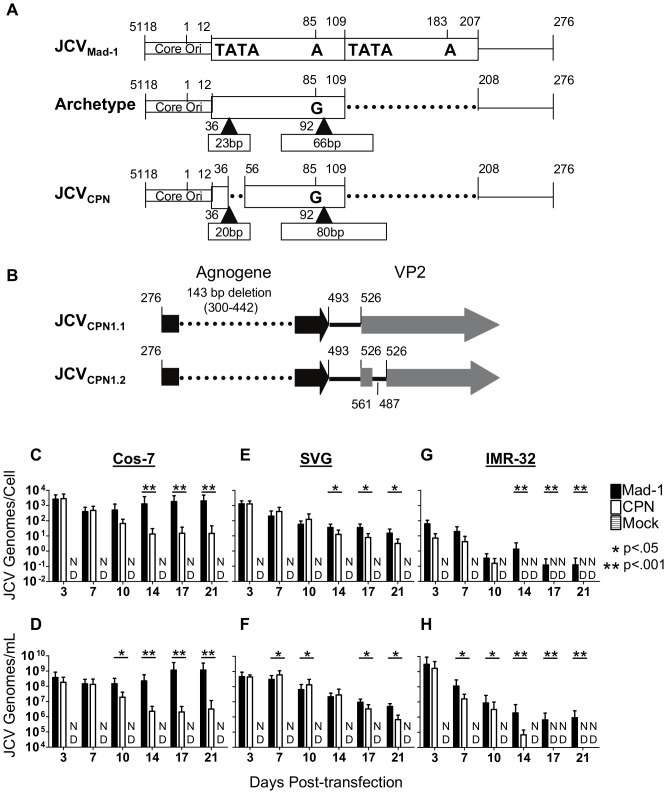
JCV_CPN_ replicates viral DNA, but at lower levels than JCV_Mad-1_. (A) JCV_CPN_ RR is archetype-like (adapted from (17)). (B) JCV_CPN1.2_ (hereafter referred to as simply JCV_CPN_) agnogene contains a 143 base pair deletion followed by a 75 base pair duplication at the beginning of the VP2 gene. (C–H) Cos-7, SVG and IMR-32 cells were transfected with linearized JCV genomes, or mock transfected. Cells were subcultured every 3–4 days and cell and supernatant samples were collected. DNA was extracted from the samples, digested with DpnI to remove input plasmid DNA, and analyzed by QPCR. Data represents the average of 4–10 independent experiments. In Cos-7 cell lysate (C) and supernatant (D) JCV_Mad-1_ but not JCV_CPN_ establishes a persistent infection. In SVG cell lysate (E) and supernatant (F) JCV_Mad-1_ and JCV_CPN_ both establish persistent infections. In IMR-32 cells, JCV_Mad-1_ infection persists for 21 days, while JCV_CPN_ becomes undetectable in cell lysate (G) and in supernatant (H). Error bars represent standard deviation. P-values were calculated using the Wilcoxon Rank Test. ND is not detected.

The JCV_CPN1_ RR is archetype-like, lacking a duplication of the TATA-box and containing a 66 bp and 80 bp insert, which contain portions of the 23 bp and 66 bp inserts present in archetype RR (Fig. 1A) [17]. A 143 bp deletion in the agnogene is also present in JCV_CPN1_ (Fig. 1B) [17]. This deletion creates a premature stop codon, and is predicted to code for a truncated 10 amino acid agnoprotein. Multiple forms of JCV_CPN_ were identified, including JCV_CPN1.1_ and JCV_CPN1.2_ which both contain the same regulatory region and agnogene deletion. In addition, JCV_CPN1.2_ contains a 75 bp duplication between the agnogene and the VP2 gene, encompassing the start of the VP2 gene (Fig. 1B). This form is predicted to encode for both a truncated and a full length VP2. Our studies determined that JCV_CPN1.1_ and JCV_CPN1.2_ are phenotypically equivalent in our cell cultures model (data not shown). Therefore, we chose to conduct the experiments in this study using the JCV_CPN1.2_ strain, because it was the predominant strain in the JCVE patient's brain. JCV_CPN1.2_ is referred to as JCV_CPN_ for the remainder of this work.

### JCV_CPN_ can replicate DNA in cell culture, but at a lower level than JCV_Mad-1_

To determine the cellular tropism of JCV_CPN_, we studied its replication in cell culture. We used multiple cell lines to model the replication of the virus in three different types of cells, kidney cells, glial cells and neurons. Cos-7 cells are African Green Monkey kidney cells which express SV40 T Ag, and are known to replicate JCV well [32]. SVG cells are human fetal glial cells, also transformed with SV40 T Ag [33]. IMR-32 cells are a human neuroblastoma cell line, derived from a tumor isolated from the abdominal cavity of a child, which do not express any polyomavirus T Ag [34]. We first wanted to determine if JCV_CPN_ is capable of genome replication. To do so, linearized genomes of JCV_CPN_ and JCV_Mad-1_ were transfected into Cos-7, SVG and IMR-32 cells. DNA from cell lysate, representing JCV DNA replicated in the transfected cells, and supernatant, representing DNA released from the transfected cells, was extracted and digested with DpnI to remove any remaining input plasmid DNA. JCV genome copy number was determined by QPCR. We found that JCV_CPN_ replicated DNA after transfection in all 3 cell lines tested (Fig. 1C–H). However, the levels of DNA were not equivalent to those seen with the prototype strain JCV_Mad-1_.

In Cos-7 kidney cells, JCV_Mad-1_ establishes a high level infection that persists over 3 weeks, as measured by copies JCV DNA in the cell lysate and supernatant (Fig. 1C and 1D). JCV_CPN_ has equivalently high levels of JCV genomes present in the cell lysate and supernatant during the first week post-transfection, but does not maintain such a high viral load, with the copy number decreasing with time. In SVG glial cells, both JCV_Mad-1_ and JCV_CPN_ establish persistent infections (Fig 1E and 1F). Levels of JCV_CPN_ genome copies in cell lysate and supernatant at later time points post-transfection are significantly lower than those of JCV_Mad-1_, but with a smaller magnitude of difference than observed in Cos-7 cells. In IMR-32 neuronal cells, JCV_Mad-1_ DNA decreased over the first 2 weeks after transfection, and then leveled off, remaining detectable over 3 weeks (Fig. 1G and 1H). In contrast, JCV_CPN_ decreased and dropped below the limit of detection at 14 days post-transfection in cell lysate and 17 days post-transfection in the supernatant. These results indicate that JCV_CPN_ is able to replicate its genome after transfection into Cos-7, SVG and IMR-32 cells, but may have a decreased capacity to persist and spread within the culture compared to prototype JCV_Mad-1_. Additionally, JCV_CPN_ does not display a replication advantage in the neuronal cell culture line tested.

### JCV_CPN_ expresses both early and late transcripts, but at decreased levels compared to JCV_Mad-1_

**Figure 2:**
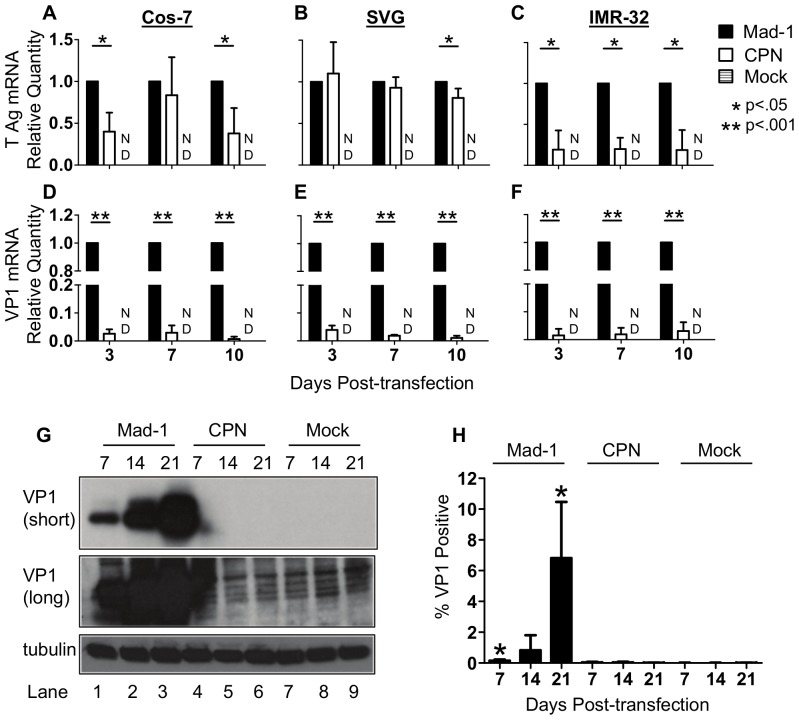
JCV_CPN_ expresses less early and late mRNA and VP1 protein than JCV_Mad-1_. (A–F) Cos-7, SVG and IMR-32 cells were transfected with JCV_Mad-1_, JCV_CPN_ or mock transfected and samples collected as described in Figure 1. qRT-PCR was used to determine the levels of early (T Ag) and late (VP1) transcripts. Relative Quantity (RQ) was calculated using the ΔΔCt method, using TATA-Box Binding Protein (TBP) as the endogenous control and JCV_Mad-1_ as the calibrator sample. Data represents the average of 5–6 independent experiments. (A and D) In Cos-7 cells, JCV_CPN_ expresses significantly lower levels of T Ag (A) and VP1 (D) mRNA. (B and E) In SVG cells, levels of T Ag (B) mRNA expressed by JCV_CPN_ are similar to JCV_Mad-1_, while VP1 (E) mRNA is significantly lower. (C and F) JCV_CPN_ expresses significantly less T Ag (C) and VP1 (F) mRNA in IMR-32 cells. Error Bars represent standard deviation. P values were calculated for Student's t test using univariate analysis. ND is not detected. (G) Western blots were done with PAB597 (anti-VP1) using cell lysate from Cos-7 cells transfected with either JCV_Mad-1_ (Lanes 1–3), JCV_CPN_ (Lanes 7–9) or mock transfected cells (Lanes 4–6) collected 7 (Lanes1,4,7), 14 (Lanes 2,5,8) or 21 (Lanes 3,6,9) days post-transfection. VP1 can be detected in JCV_Mad-1_ transfected cells at all time points, but not at any time with JCV_CPN_, with either a short (upper panel) or long (middle panel) exposure. Anti-tubulin antibody was used for loading control (lower panel). Blots are representative of 3 independent experiments (H) JCV_Mad-1_, JCV_CPN_ or mock transfected Cos-7 cells were analyzed for VP1 expression by flow cytometry. JCV_Mad-1_ but not JCV_CPN_ transfected cells have significantly higher levels of VP1 positive cells than Mock transfected samples. Results are the average of 4 independent experiments. Error bars represent standard deviation. P values were calculated using students t test, comparing JCV_Mad-1_ and JCV_CPN_ to mock.

Knowing that JCV_CPN_ is able to replicate viral DNA, but not establish a persistent high level infection, we wanted to determine if JCV_CPN_ is able initiate transcription of mRNAs from the early and late promoters. To do so, we used qRT-PCR on RNA from transfected cells using primer and probe sets located in T Ag to detect early transcripts, and in VP1 to detect late transcripts. In Cos-7, SVG and IMR-32 cells, JCV_CPN_ expresses detectable levels of both the early and late transcripts (Fig. 2). In Cos-7 cells, the level of early transcripts is significantly lower than those seen with JCV_Mad-1_ 3 and 10 days post-transfection (Fig. 2A). In SVG cells, the level of early transcripts is similar between JCV_CPN_ and JCV_Mad-1_ 3 and 7 days post-transfection, and significantly lower 10 days post-transfection (Fig. 2B). In IMR-32 cells, level of early transcripts produced by JCV_CPN_ is significantly lower than those of JCV_Mad-1_ at 3, 7 and 10 days post-transfection (Fig. 2C). In all three cell lines, the level of late transcripts is markedly lower in JCV_CPN_ transfected cells compared to JCV_Mad-1_ transfected cells (Fig. 2D–F). These results indicate that, although both the early and late promoter of JCV_CPN_ are transcriptionally active, they function at a level lower than JCV_Mad-1_. The decrease in transcript levels is larger for the late than the early promoter.

### JCV_CPN_ fails to produce detectable levels of VP1 protein in Cos-7 cells

We then wanted to determine if JCV_CPN_ produces VP1 protein from the late mRNA. JCV_Mad-1_ transfected cells produce VP1 protein, with the amount of VP1 present increasing with later times post-transfection (Fig. 2G). JCV_CPN_ does not produce detectable levels of VP1 protein, even with long exposure times (Fig. 2G).

To determine if there is VP1 present in a small number of JCV_CPN_ transfected cells, which cannot be detected by Western Blot, we developed a protocol to analyze transfected Cos-7 cells for VP1 expression using flow cytometry. JCV_Mad-1_ transfected cells were positive for VP1 expression, with an increase in the percentage of cells positive for VP1 over time, with the highest percentage of VP1 positive cells seen 21 days post-transfection (Fig. 2H). The percentage of JCV_Mad-1_ VP1 positive cells is significantly higher than the background observed in Mock transfected cells. JCV_CPN_ transfected cells are not positive for VP1 by flow cytometry at a level significantly higher than Mock transfected cells (Fig 2H). This supports the results seen in using Western blotting, that JCV_CPN_ does not express detectable levels of VP1 protein.

### JCV_CPN_ produces low levels of infectious virions

Although we could not detect VP1 expression by Western Blot or flow cytometry in transfected cells, there may be a very low level of expression below our limit of detection. We therefore wanted to determine if JCV_CPN_ transfected cells are producing virions capable of infecting a new round of cells. To do so, supernatant was collected from transfected Cos-7, SVG and IMR-32 cells 7, 14 and 21 days post-transfection and was used to infect Cos-7 cells. Infection was allowed to proceed for 7 days. Cells were collected, and analyzed for JCV DNA by QPCR or stained for VP1 and analyzed by flow cytometry.

**Figure 3:**
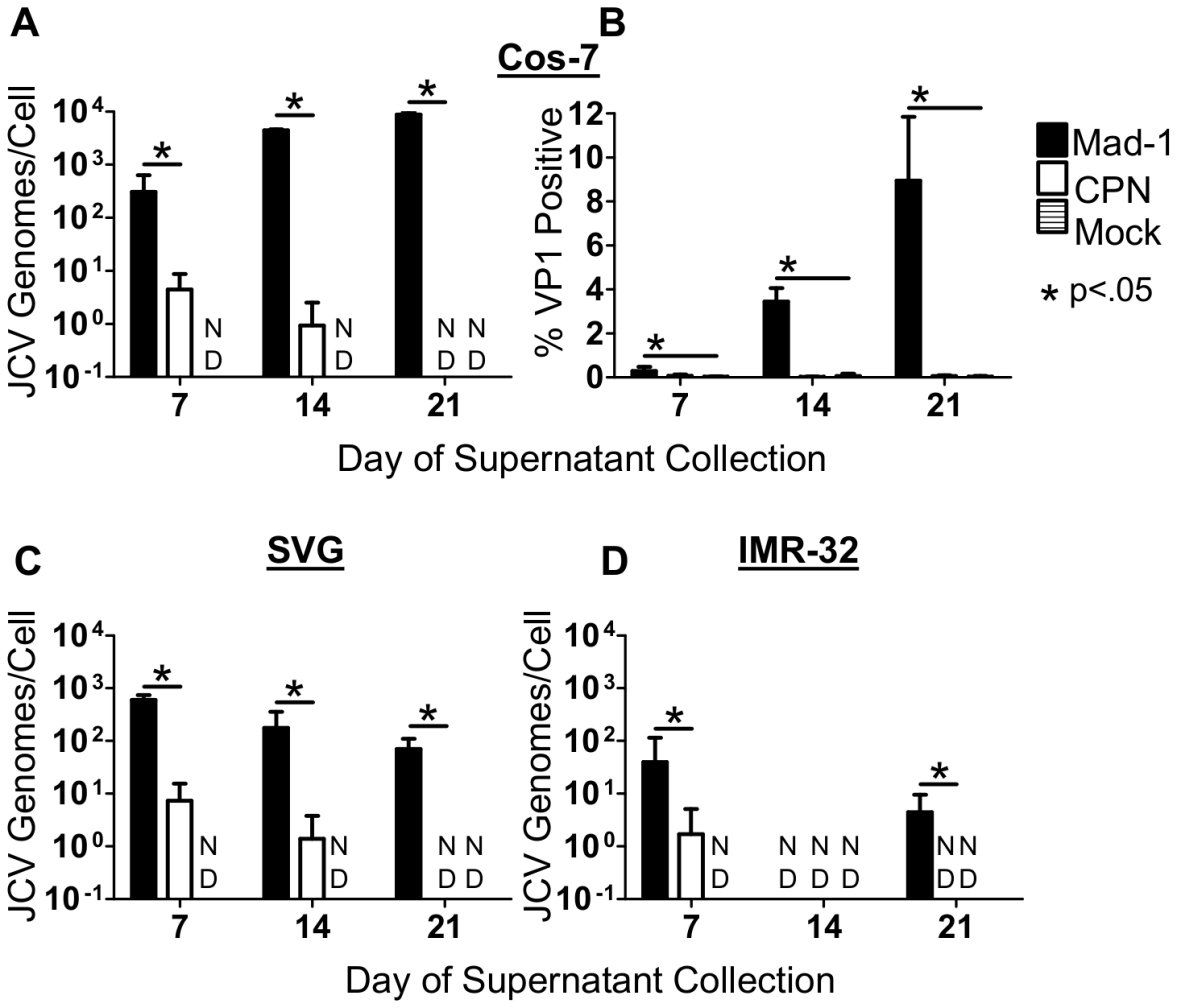
JCV_CPN_ transfected IMR-32, SVG and Cos-7 cells produce low levels of infectious virions. Supernatant from transfected Cos-7, SVG and IMR-32 cells was collected 7, 14 and 21 days post-transfection, and used to infect naive Cos-7 cells. At 7 days post-infection, cells were collected and either analyzed for JCV DNA using QPCR (A, C and D) or stained for VP1 and analyzed using flow cytometry (B). Supernatant collected 7 days post-transfection from JCV_CPN_ transfected Cos-7 (A), SVG (C) or IMR-32 (D) cells can establish an infection in naïve Cos-7 cells, as measured by the presence of JCV DNA 7 days post-infection. Levels of DNA detected with JCV_CPN_ infection are significantly lower than with JCV_Mad-1_ infection (A, C and D). (B) VP1 positive cells are detected after infection with JCV_Mad-1_, but not JCV_CPN_ containing supernatant. Data is the average of 3–4 independent experiments. Error bars represent standard deviation. P vales were calculated using Wilcoxon rank test for QPCR data, comparing JCV_CPN_ to JCV_Mad-1_. P-values for flow cytometry data were calculated using students t test, comparing JCV_Mad-1_ and JCV_CPN_ to mock at each time point. ND is not detected.

JCV_Mad-1_ containing supernatant from Cos-7 cells was able to establish an infection in the new cells, with the viral load and the percentage of cells infected increasing with supernatant collected at later time points post-transfection (Fig. 3A and B). The percentage of VP1 positive cells was significantly higher than in the samples treated with supernatant from mock transfected cells (Fig. 3B). In contrast, cells infected with JCV_CPN_ containing supernatant had significantly lower levels of viral DNA detected, which decreased over time, becoming undetectable in samples infected with supernatant collected 21 days post transfection (Fig. 3A). The percentage of JCV_CPN_ VP1 positive cells was not significantly higher than observed with Mock supernatant (Fig. 3B), which is most likely due to the lack of detectable expression of VP1 protein in JCV_CPN_ transfected cells.

Similar results were seen using supernatant from transfected SVG and IMR-32 cells, with low levels JCV DNA detected in cells infected with JCV_CPN_ containing supernatant collected 7 days post-transfection, and then decreasing (Fig. 3C and D). Using supernatant from SVG cells, JCV_CPN_ DNA remains detectable with supernatant collected 14, but not 21, days post-transfection and JCV_Mad-1_ infected cells had DNA levels that were significantly higher than those observed with JCV_CPN_ at all time points (Fig. 3C). In contrast to the results observed using Cos-7 supernatant, the viral loads in JCV_Mad-1_ infected cells decreased with later collection points using SVG supernatant (Fig. 3C). Cells infected with supernatant collected 14 and 21 days post-transfection from IMR-32 cells had undetectable JCV_CPN_ DNA levels (Fig. 3D). JCV_Mad-1_ DNA was detected in cells infected with supernatant collected from IMR-32 cells at 7 and 21 days, but not 14 days, post-transfection (Fig. 3D). This is most likely due to the levels of JCV DNA being below the limit of detection of our assay.

### Generation of chimeras and agnogene mutants

**Figure 4:**
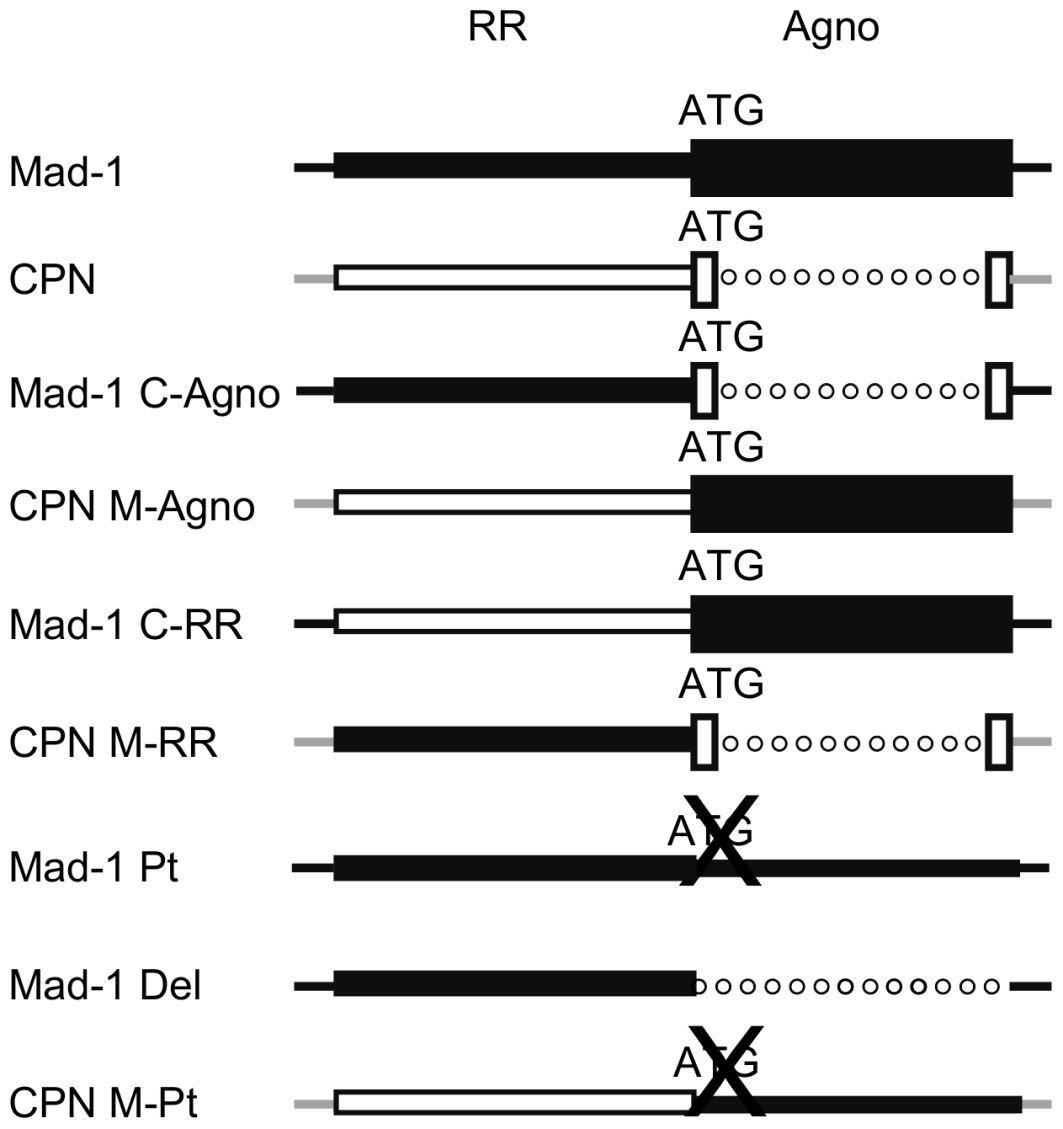
JCV_Mad-1_ and JCV_CPN_ chimeras and agno deletion mutants. This diagram shows the various chimeric viruses and deletion mutants generated. Black represents sequences from JCV_Mad-1_ and white represents sequences from JCV_CPN_. Lines represent DNA sequences. Dotted Lines represent deletions. Boxes represent genes. An X through ATG represents mutation of the start codon to eliminate protein expression. Mad-1 C-Agno is JCV_Mad-1_ with the JCV_CPN_ agnogene introduced. CPN M-Agno is JCV_CPN_ with a full length agnogene from JCV_Mad-1_ introduced. Mad-1 C-RR is JCV_Mad-1_ with the JCV_CPN_ RR and CPN M-RR is JCV_CPN_ with the JCV_Mad-1_ RR. Mad-1 Pt is JCV_Mad-1_ with a mutated start codon which prevents the expression of the agnoprotein and Mad-1 Del is JCV_Mad-1_ with the entire agnogene deleted. CPN M-Pt is JCV_CPN_ with the agnogene of Mad-1 Pt.

The results of the above experiments comparing JCV_CPN_ and JCV_Mad-1_ suggest that JCV_CPN_ has a block preventing late gene expression and protein production, as well as in the production and release of infectious virions. The two major regions of difference between JCV_CPN_ and JCV_Mad-1_ are the RR and the agnogene. To determine which area of the virus is the major contributor to the phenotype of JCV_CPN_, we generated chimeric viruses of JCV_CPN_ and JCV_Mad-1_ (Fig. 4). We swapped the agnogene genes of the two viruses to generate Mad-1 C-Agno and CPN M-Agno. Mad-1 C-RR and CPN M-RR were generated by exchanging the RRs of the two viruses. We obtained two agno deletion mutations, Mad-1 Pt, which has a start codon point mutation which prevents the expression of agnoprotein, and Mad-1 Del, which has the entire agnogene deleted. CPN M-Pt contains the full length agnogene with the start codon point mutation from Mad-1 Pt. We studied these additional mutants in Cos-7 cells, because these cells displayed the greatest difference in phenotype between JCV_Mad-1_ and JCV_CPN_.

### The agnogene deletion is the major contributor to the phenotype of JCV_CPN_

**Figure 5:**
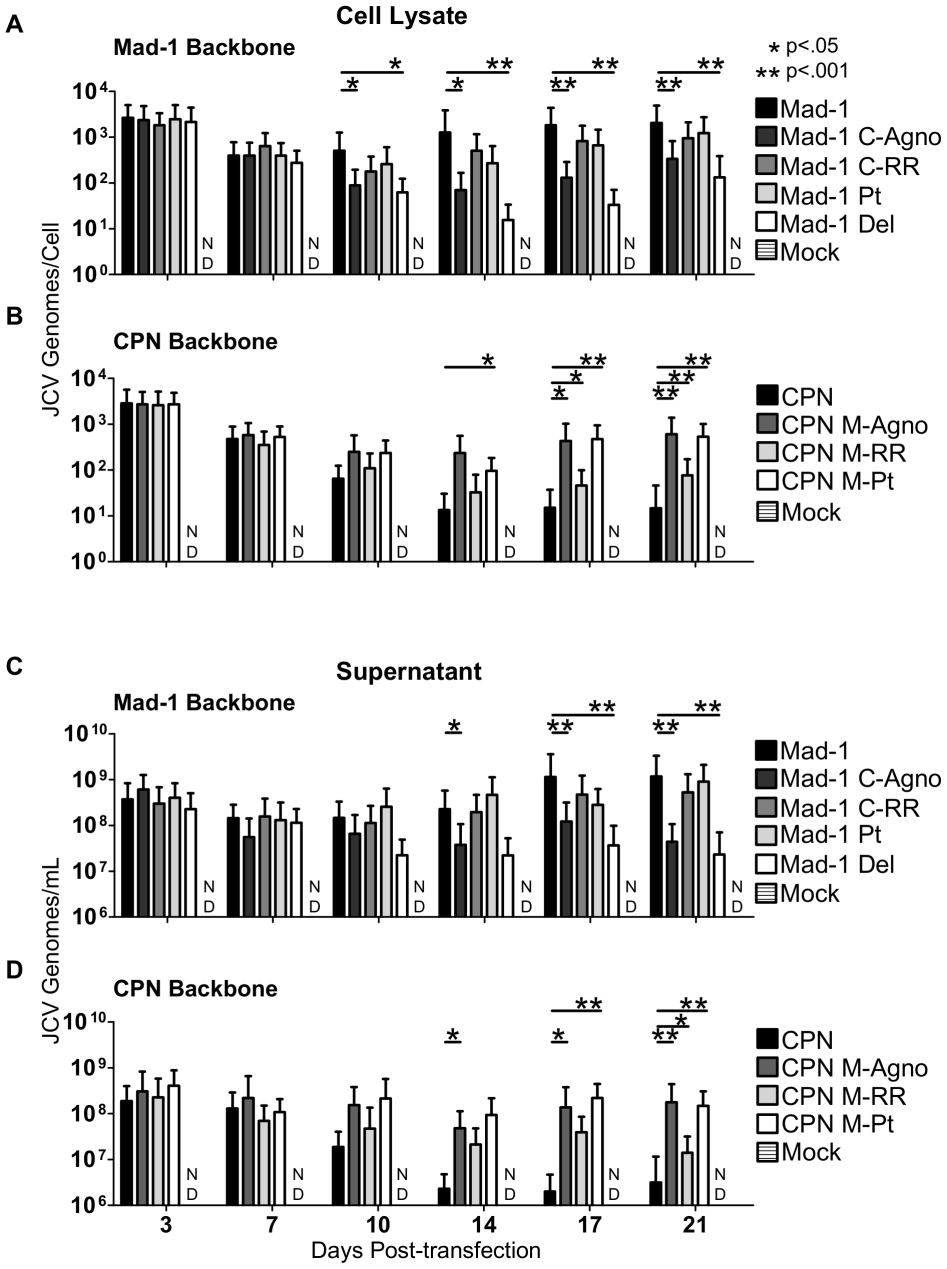
The agnogene deletion of JCV_CPN_ is the primary cause of its replication defect. Linearized JCV DNA of JCV_Mad-1_, Mad-1 C-Agno, Mad-1 C-RR, Mad-1 Pt, Mad-1 Del, JCV_CPN_, CPN M-Agno, CPN M-RR, and CPN M-Pt were transfected into Cos-7 cells and DNA levels were quantified over 3 weeks, as described in Figure 1. (A and C) Levels of DNA with JCV_Mad-1_ and the chimeras and agno deletion viruses on the JCV_Mad-1_ backbone were measured in the cell lysate (A) and supernatant (C). Mad-1 C-Agno and Mad-1 Del show significantly lower levels of DNA late in infection. (B and D) Levels of DNA with JCV_CPN_ and the chimeras and agno deletion viruses on the JCV_CPN_ backbone were measured in the cell lysate (B) and supernatant (D). Introduction of a full length agnogene causes the greatest increase in DNA levels. Data represents the average of 4–10 independent experiments. Error bars represent standard deviation. P-values were calculated with the Wilcoxon Rank Test. ND is not detected.

To begin characterizing the phenotypes of the viruses shown in Figure 4, linearized DNA was transfected into Cos-7 cells, and JCV DNA levels in cell lysate and supernatant were monitored for 3 weeks using QPCR (Fig. 5). Mad-1 C-Agno has significantly lower levels of DNA from 10–21 days post-transfection compared to Mad-1, while Mad-1 C-RR has DNA levels similar to Mad-1 (Fig. 5A and 5C). This indicates that introducing the agnogene deletion of JCV_CPN_ into Mad-1 results in a decrease in DNA replication. In contrast, the introduction of the JCV_CPN_ RR does not. This suggests that the major cause of the replication kinetics seen with JCV_CPN_ are due to the agnogene deletion, and the not the archetype-like RR. When comparing the agno deletion viruses to JCV_Mad-1_, Mad-1 Pt has similar DNA levels, while Mad-1 Del had decreased levels late in infection similar to JCV_CPN_ (Fig. 5A and 5C). This provides evidence the gene deletion is more important than the loss of the agnoprotein for the observed phenotype.

Furthermore, the results seen with the viruses on the JCV_CPN_ backbone support these conclusions. CPN M-Agno, with the full length agnogene and agnoprotein, and CPN M-Pt, with just a full length agnogene, have the greatest increase of JCV DNA levels compare to JCV_CPN_, and in both cases the level of DNA increase is similar (Fig. 5B and 5D). This supports the conclusion that the deletion in the agnogene causes the decreased replication ability of JCV_CPN_. CPN M-RR, with the JCV_Mad-1_ RR, also show some increase in DNA levels, but to a lesser extent than CPN M-Agno (Fig. 5B and 5D).

### Deletion in the agnogene prevents expression of VP1 protein

**Figure 6:**
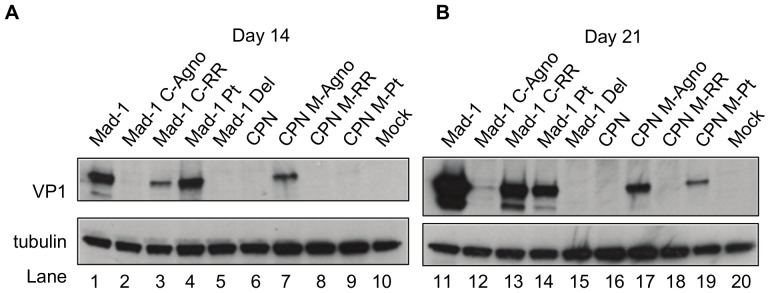
Deletion in the agnogene prevents VP1 expression. Western blots for VP1 were done as described in Figure 2. (A) VP1 levels in Cos-7 cell lysate 14 days post-transfection. (B) VP1 levels in Cos-7 cells 21 days post-transfection. Levels of VP1 expression are drastically reduced in Mad-1 C-Agno, and expression is rescued by the full length agnogene in CPN M-Agno. Deletion of the agnogene Mad-1 Del results in a greater decrease in VP1 expression than just the prevention of the protein expression in Mad-1 Pt. Blots are representative of 3 or 4 independent experiments.

We then sought to determine if the levels of VP1 protein expression would correspond with levels of viral DNA in cells transfected with the chimeras and agno deletion mutants. Western blots were done for VP1 expression in JCV transfected Cos-7 cells. At 14 days post-transfection, only JCV_Mad-1_, Mad-1 Pt, Mad-1 C-RR and CPN M-Agno have detectable levels of VP1 (Fig. 6A). At 21 days post-transfection, all of these viruses and CPN M-Pt have detectable VP1 protein expression (Fig. 6B). All of these viruses have full length agnogenes, but Mad-1 Pt does not have agnoprotein expression. Compared to JCV_Mad-1_, Mad-1 Pt shows some decrease in VP1 expression, while Mad-1 Del has a complete lack of VP1 expression. All of the viruses with the JCV_CPN_ agnogene deletion, JCV_CPN_, Mad-1 C-Agno and CPN M-RR lack VP1 expression (Fig. 6A and 6B). Taken together, these results indicate that the deletion in the agnogene results in decreased or undetectable levels of VP1 protein expression, and that the levels of DNA replication correspond with the presence of VP1 protein expression.

### Deletions in the agnogene reduce the production of infectious virions

**Figure 7:**
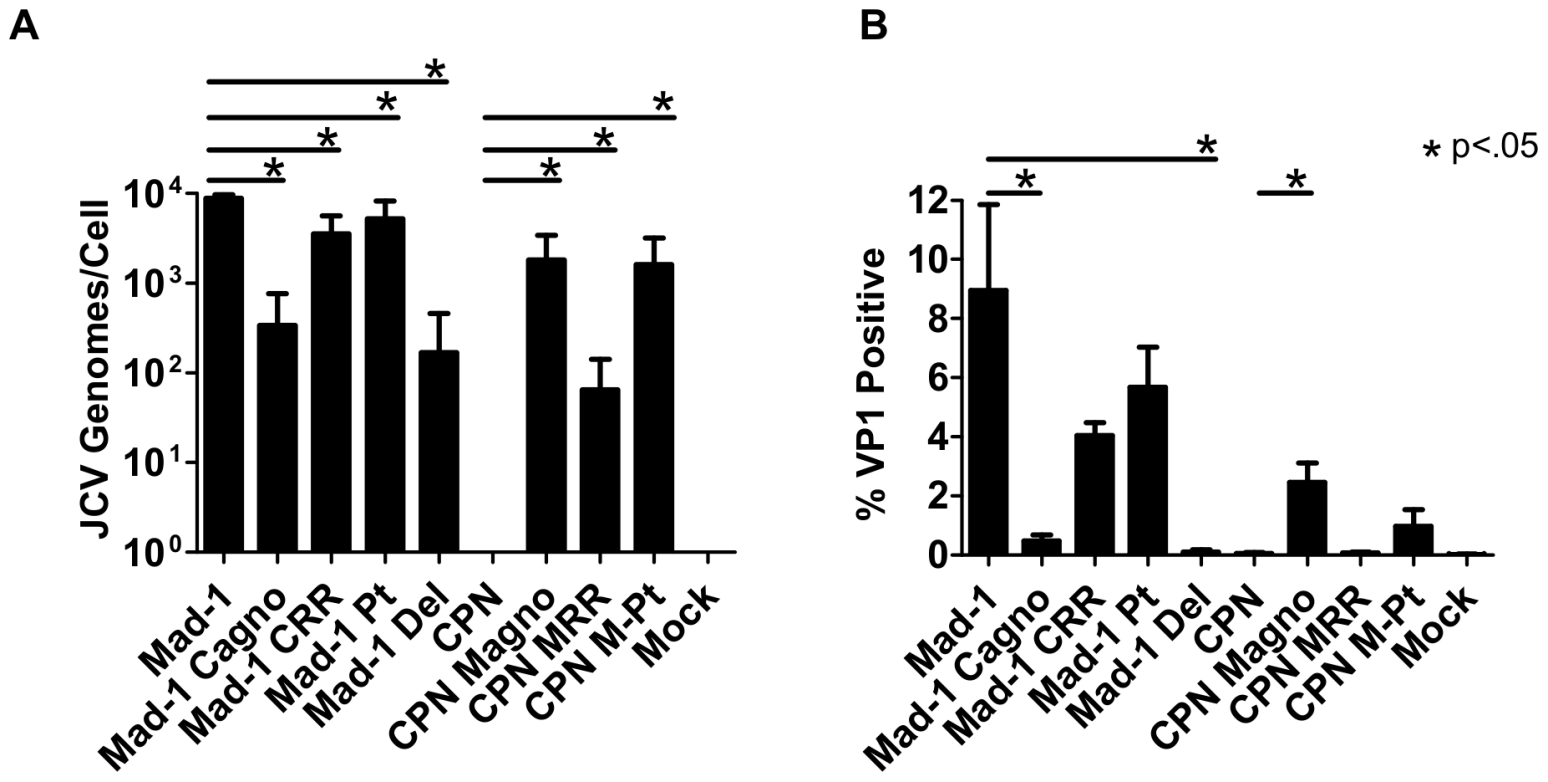
Loss of the agnogene results in decreased production of infectious virions. Supernatant was collected from Cos-7 cells 21 days post-transfection and used to infect naive Cos-7 cells. After 7 days, the cells were analyzed for (A) JCV DNA by QPCR or (B) VP1 expression by flow cytometry. Deletions in the agnogene in JCV_Mad-1_-infected cells resulted in a significant decrease in JCV genomes/cell and percentage of cells expressing VP1, while introduction of a full length agnogene into JCV_CPN_ results in a significant increase in the viral load and percentage of cells expressing VP1. Data is the average of 3 independent experiments. Error bars represent standard deviation. P values were calculated using Wilcoxon rank test for QPCR data, comparing each virus to its parent virus, either JCV_Mad-1_ or JCV_CPN_. P-vales for flow cytometry data were calculated using students t test using the same sets of comparisons. ND is not detected.

Finally, we sought to determine if the ability to produce infectious virions of the chimeric and agno deletion viruses also corresponds with DNA levels and VP1 protein production in these cells. Supernatant collected from Cos-7 cells 21 days post-transfection was used to infect naïve Cos-7 cells, and the DNA levels (Fig. 7A) and percentage of cells expressing VP1 (Fig. 7B) were determined by QPCR and flow cytometry, respectively, at day 7. Compared to JCV_Mad-1_-infected cells, both Mad-1 C-agno and Mad-1 Del –infected cells have significantly lower viral loads, showing a 1.5–2 log decrease (Fig. 7A). Mad-1 Pt and Mad-1 C-RR-infected cells also have significantly lower viral loads than JCV_Mad-1_-infected cells, but tended to have a smaller magnitude of decrease (Fig. 7A). Mad-1 C-agno and Mad-1 Del-infected cells have significantly lower percentages of cells expressing VP1, while Mad-1 Pt and Mad-1 C-RR-infected cells do not (Fig. 7B). CPN M-Agno, CPN M-RR and CPN M-Pt-infected cells all have detectable DNA levels, with significantly higher viral loads compared to JCV_CPN_ (Fig. 7A). Compared with JCV _CPN_-infected cells, CPN M-Agno-infected cells have a significantly higher percentage cells expressing VP1, and CPN M-Pt-infected cells tended to have more cells expressing VP1. Interestingly, introduction of only the full length gene without protein expression in CPN M-Pt is enough to rescue the DNA levels, and show some increase in percent of cells expressing VP1.

The results of these infection experiments suggest that the deletion in the agnogene is the primary cause of the phenotype observed with JCV_CPN_. However, CPN M-RR, with the JCV_Mad-1_ RR does show some increase in DNA replication and production of infectious virions compare to JCV_CPN_, which may indicate that the RR composition is also affecting replication of the virus, but to a lesser degree than the agnogene deletion. It is likely that the combination of the agnogene deletion together with archetype-like RR is the cause of the overall phenotype of JCV_CPN_. Overall, these experiments have used the unique naturally occurring JCV_CPN_ agnogene deletion and archetype-like RR to clarify the role of the agnogene and RR forms in JCV replication in cell culture.

## Discussion

JCV_CPN_ was isolated from the brain of the first patient to be diagnosed with JCVE, a novel syndrome characterized by infection of cortical pyramidal neurons. JCV_CPN_ is the first naturally occurring isolate with a large deletion in the agnogene, which was originally called agno due to the unknown nature of its function. While studies have begun to shed light on the function of the agnoprotein and agnogene, there is still much to be learned. JCV_CPN_ presents the opportunity to study the function of the agnogene and agnoprotein in a naturally occurring pathogenic variant of JCV, isolated in association with infection of a new cell type.

In this study, we compared the replication of JCV_CPN_ to that of the prototype virus JCV_Mad-1_. We used Cos-7 cells to model infection in kidney cells and SVG cells as a glial cell model. Both of these cell lines are widely used to study the replication of JCV in kidney and glial cells, and it is commonly thought that the results obtained in them are applicable to what would occur in these cell types during JCV infection in humans. Studying the replication of JCV in cortical pyramidal neurons is challenging, as there are no cortical pyramidal neuron cell lines available. We therefore used IMR-32 neuroblastoma cells to model neuronal infection. Using these cell lines allowed us to study the replication of JCV_CPN_ in different cell types in cell culture. We found that JCV_CPN_ was capable of replicating its genome in cell culture. However, it could not establish an infection at the same level observed with JCV_Mad-1_ over time and did not display any replication advantage over JCV_Mad-1_ in any cell line. Levels of JCV_CPN_ DNA replication in Cos-7 and IMR-32 cells were similar to JCV_Mad-1_ at early time points, and then decreased at later time points, indicating that the virus is able to replicate viral DNA after transfection but the infection could not spread and persist in the cell culture at the same level as JCV_Mad-1_. In addition, mRNA and VP1 protein analyses showed some decrease in early transcription and a marked decrease in late transcription, with no detectable VP1 protein production. In all three cell lines, low levels of infection were observed using supernatant from JCV_CPN_ transfected cells, but only using supernatant collected at early times post-transfection, and at levels significantly lower than seen with JCV_Mad-1_. These data indicate that the virus has a block in late gene expression and protein production, which results in low levels of infectious virions being released, and an inability to sustain a persistent high level infection in cell culture. Furthermore, since agnoprotein has been implicated in late stages of viral maturation, there may be an additional block at the level of virion formation and/or release independent of the lack of VP1 production.

JCV_CPN_ and JCV_Mad-1_ are different in two regions, the RR and the agnogene. This provided us with the unique opportunity to determine for the first time how each of these regions contributed to the phenotype of JCV_CPN_. We therefore studied a series of chimeric viruses of the RR and agnogene. Interestingly, the agnogene deletion in JCV_CPN_ was the predominant cause of its phenotype, not the archetype-like RR. Moreover, the loss of the agnogene DNA had a larger effect on the replication of the virus than the loss of agnoprotein expression. Finally, the archetype-like RR also showed a detrimental effect on JCV_CPN_ replication, but of a smaller magnitude than that of the agnogene deletion. For these experiments we used Cos-7 cells, because they displayed the largest difference in phenotype between JCV_CPN_ and JCV_Mad-1_ and allowed us to use the greatest number of techniques to study these viruses. We believe that the results obtained in these experiments are representative of what would be observed with the other cell lines used in this study.

Previous studies have implied that agnoprotein had a role in viral DNA replication [23], [24]. However, JCV_CPN_ is able to produce levels of DNA similar to JCV_Mad-1_ early after transfection, with DNA levels decreasing with time. These results suggest that an agnogene-deletion mutant can indeed replicate DNA, but levels drop off due to blocks at later steps in infection, thereby preventing spread within the cell culture. Conflicting studies have associated agnoprotein with gene expression, with both the loss of agnoprotein [19], [30] or its presence [23], [26] suppressing activity of the late promoter of JCV in different experimental systems. Additionally, deletion of the full agnogene causes a greater decrease in VP1 expression than prevention of agnoprotein expression without deletion of the agnogene DNA [30]. Our data shows that the deletion in the agnogene of JCV_CPN_ results in a block in late gene expression, impairing the release of virions capable of infecting new cells and propagating the infection in cell culture. This is consistent with the results of Akan et al [30] and highlights the importance of the agnogene DNA in addition to the protein it codes for. Further studies are needed to determine the mechanism of the block in VP1 expression. One possibility is that a host cell factor which binds to one of the sites in the agnogene, identified by Akan et al [30], is involved in expression of the late transcript. Further investigations to identify these cellular proteins are warranted to determine the mechanism by which they act during JCV replication. Another potential mechanism is that deletions in the agnogene somehow alter the splicing or translation of VP1 from the late mRNA.

Previous studies of JCV deletion mutants lacking agnoprotein expression have implicated the agnoprotein in genome packaging and/or virion formation and release, with empty capsids being produced by viruses lacking the agnoprotein [27], [28]. In our study however, the agnoprotein start codon point mutant was able to replicate its DNA and produce infectious virions at a level similar to JCV_Mad-1_in Cos-7 cells, which suggests that at least in these cells, the agnoprotein is not necessary for formation and release of virions containing viral genomes.

Archetype RR is typically found in the urine, but the JCV_CPN_ RR isolated from the brain of the JCVE patient is archetype-like. Studies of the replication of different forms of RR have shown that archetype RR form has less active early and late promoters [36]. Surprisingly, our study did not find the archetype-like RR of JCV_CPN_ to be the primary cause of the decreased levels of late transcription and VP1 protein expression. Introducing the archetype-like RR of JCV_CPN_ into JCV_Mad-1_ had little effect on the ability of the virus to replicate. JCV_Mad-1_ RR introduced into JCV_CPN_ partially rescues replication, but had a lesser impact than introduction of a full length agnogene. In fact the archetype-like RR only has a significant impact on the virus once it has already been impaired by a deletion in the agnogene, indicating that not all archetype-like RR forms found in nature lead to lower levels of viral replication.

While JCV_CPN_ was unable to establish a persistent infection in our cell culture model, it was found at high levels in the brain of the JCVE patient. JCV_CPN_ does produce some infectious virions in cell culture, but at much lower levels than JCV_Mad-1_. This does not, however, mean that the virus was unable to establish an infection in the brain of the JCVE patient. There are limitations to the cell culture model we utilized in this study. First, the infection in a person can take place on the timescale of years to decades, while our experiments were done over weeks. Second, this individual was also infected with a strain of JCV with a full length agnogene and agnoprotein, which may have influenced the replication of JCV_CPN_. Third, it may be that having low levels of replication in the brain was actually advantageous to the virus while faced with a healthy immune system, allowing it to accumulate until the immune system became compromised and JCVE developed.

After observing the infection of granule cell neurons in JCV GCN and cortical pyramidal neurons in JCVE, we conducted studies to determine if infection of neurons by JCV was limited to these syndromes, or if it is more widespread. Based on staining of PML brain samples, it is predicted that up to 51% of patients have granule cell neurons infected by JCV [37]. Additionally, infection of cortical neurons by JCV in classic PML cases has been observed with infection present in the gray white junction and gray matter [38]. These studies demonstrate that infection of neurons occurs in a large number of PML patients, and is not limited to patients with JCV GCN or JCVE. Further studies of the molecular composition of JCV in neurons of patients with classic PML are needed to determine whether these cells are also infected by JCV-deletion mutants.

The study of unique pathogenic isolates of JCV, such as JCV_CPN_ or JCV_GCN_ allows us to decipher the basic biology of JCV replication using mutations that have arisen naturally during infection in humans. Our studies of JCV_CPN_ have provided valuable insights into both the function of the agnogene and agnoprotein, as well as a naturally occurring variation of the regulatory region, on JCV replication. Our results have helped clarify the role of the agnogene and agnoprotein in DNA replication, transcription and protein expression, demonstrating that a deletion in the agnogene has a dramatic effect on the expression of VP1 protein and the production of infectious virions. Further studies of naturally occurring variants of JCV will continue to add clarity to our understanding of the biology of JCV replication and pathogenesis.
